# The impact of N-acetylcysteine and ascorbic acid in contrast-induced nephropathy in critical care patients: an open-label randomized controlled study

**DOI:** 10.1186/s13054-017-1862-3

**Published:** 2017-10-31

**Authors:** Eleni Palli, Demosthenes Makris, John Papanikolaou, Grigorios Garoufalis, Irini Tsilioni, Paris Zygoulis, Epaminondas Zakynthinos

**Affiliations:** Department of Critical Care, University Hospital of Larissa, Thessaly, Greece

**Keywords:** Contrast induced nephropathy, Intensive care, N-acetylcysteine, Ascorbic acid, 8-Isoprostane, cystatin-C

## Abstract

**Background:**

The aim was to investigate whether the use of N-acetylcysteine and ascorbic acid reduce contrast-induced nephropathy incidence in critical care patients.

**Methods:**

This was a one-center, two-arm, prospective, randomized, open-label, controlled trial in the Intensive Care Unit of the University Hospital of Larissa, Greece. Patients with stable renal function, who underwent non urgent contrast-enhanced computed tomography for diagnostic purposes, were included in the study. Patients in the treatment group (NacA, n = 60) received intravenously N-acetylcysteine (1200 mg) and ascorbic acid (2 g) dissolved separately in 100 ml of normal saline 2 hours before, and at 10 hours and 18 hours following the infusion of contrast agent, while control group patients (CG, n = 64) received only normal saline. All patients received additional hydration. Contrast-induced nephropathy was defined as relative increase by 25% of the baseline values of serum creatinine.

**Results:**

Contrast-induced nephropathy in NacA and CG were 18.33% and 15.6%, respectively (*p* = 0.81). The percentage change median (interquartile range (IR)) of serum cystatin-C (mg/L) from baseline in patients who underwent contrast-induced tomography, were 37.23% (28.53) and 93.20% (46.90) in NacA and in CG, respectively (*p* = 0.03). The 8-isoprostane serum levels in NacA were significantly lower compared to CG at 2 hours (*p* = 0.012) and 24 hours (*p* = 0.006) following radiocontrast infusion. Multivariate analysis revealed that contrast-induced nephropathy was independently associated with a higher baseline ratio of serum urea/creatinine (odds ratio, 1.02; 95 CI%, 1.00–1.05) and with the use of nephrotoxic medications (odds ratio, 0.24; 95 CI%, 0.06–0.94).

**Conclusion:**

Intravenous administration of N-acetylcysteine and ascorbic acid failed to reduce contrast-induced nephropathy in critically ill patients who underwent contrast-enhanced computed tomography, despite a significant reduction of 8-isoprostane levels in treated patients.

**Trial registration:**

ClinicalTrials.gov, NCT01017796. Registered on 20 November 2009.

**Electronic supplementary material:**

The online version of this article (doi:10.1186/s13054-017-1862-3) contains supplementary material, which is available to authorized users.

## Background

Critically ill patients represent a group vulnerable to renal function deterioration due to the severity of the illness and because they are under several potential nephrotoxic hazards. Moreover, critically ill patients often have to undergo computed tomography (CT) or angiography for diagnostic/therapeutic purposes, with the use of contrast agents that may induce nephropathy - commonly known as contrast-induced nephropathy (CIN) [1]. CIN increases the need for renal replacement therapy (RRT), prolongs hospitalization and increases morbidity and mortality [2–8].

However, those data on the impact of radiocontrast material on renal function come mainly from patients with cardiac conditions who underwent coronary percutaneous procedures [2, 7–12]. Only a few studies have addressed this issue in the critical care setting. Hence, the incidence of CIN varies considerably from 1.4% to 50% depending on the characteristics of the studied population, the material and dose used and the criteria that have been used for renal impairment detection [13–15]. In a previous study we showed that in relation to their younger counterparts, older critically ill patients are more prone to developing renal dysfunction after the intravenous infusion of the contrast agent [16].

CIN has been reported to be associated with increased oxidative stress [17] and several clinical factors have been identified that increase its risk in patients with diabetes mellitus or patients with cardiac conditions [9, 10, 18]. Yet, data on the use of various protective measures in the critical care setting are limited [16, 19]. In this study we therefore aimed to investigate the impact of the combination of two antioxidant agents, N-acetylcysteine (Nac) and ascorbic acid (Aa), in critically ill patients who undergo contrast-enhanced CT. We hypothesized that the use of antioxidant agents could balance the increased oxidative burden that is associated with the use of the radiocontrast material [17].

## Methods

### Design and population

The present study is a one-center, two-arm, randomized, open-label, controlled trial. The study took place in the Intensive Care Unit (ICU) of the University Hospital of Larissa (12 beds) between 2010 and 2013. Inclusion criteria were age ≥14 years and diagnostic need for contrast-enhanced CT. Exclusion criteria were history of intravascular administration of contrast agent during the 6-day period prior to randomization, the use of antioxidant agents during the last week before the examination, unstable renal function, use of RRT in the 3-day period before randomization and pregnancy. We defined the unstable renal function as a change in serum creatinine values greater than 20% between 2 consecutive days during the 3 days prior to randomization, independently from crude baseline renal function.

### Study protocol

Patients were randomized to intravenously receive Nac (1200 mg) and Aa (2 g) dissolved separately in 100 ml of normal saline (N/S) 0.9%, 2 hours before and at 10 hours and 18 hours following the infusion of contrast agent (treatment group - NacA) or 200 ml of intravenous N/S 0.9% (control group - CG) at the same time points as NacA. The choice of Nac and Aa and their doses were based on previous studies that aimed to manage oxidative damage [9], whereas Nac presents vasodilatory properties [20] that could overcome vasoconstriction caused by the contrast agent [21].

All participants received the same amount of additional hydration with 1000 ml N/S 0.9% given intravenously before CT as protection against the intravenous constant medium, unless this was contraindicated based on concurrent hemodynamic assessment; the latter was assessed either by ultrasonography or thermo-dilution [22]. Randomization was performed using tables of random numbers.

Serum urea, creatinine concentrations were assessed before the infusion of the contrast agent and once daily until the 5^th^ day following radiocontrast infusion; serum cystatin-C assessed before and at 24 and 48 hours following radiocontrast infusion and 8-isoprostane was assessed before and at 2, 24 and 48 hours following radiocontrast infusion. Timing of renal function assessment was based on creatinine and cystatin-C expected peak serum levels following the infusion of radiocontrast agent; serum creatinine peaks at 3–5 days [23] while serum cystatin-C peaks at 1–2 days after the infusion of contrast agent [24].

CIN was defined as relative increase by 25% of serum creatinine from the baseline value within 5 days [24]. CT scans were performed according to the institute’s standard protocol with the use of the same agent, iopamidol, a low osmolarity, non ionic, iodinated contrast medium (Iopamiro 370, Bracco). The quantity of infused contrast agent was determined by the radiologists depending on the type of CT imaging, and patients related characteristics and was recorded in a dedicated chart. The dose of contrast medium for contrast-enhanced CT was generally 0.5–2 ml/Kg of body weight. The minimum used dose was 100 ml and 150 ml was the maximum dose that was given. Measurement of serum cystatin-C and 8-isoprostane were performed with commercial enzyme-linked immunoassay kits (Cayman CC, USA).

### Outcomes

The main outcome was the incidence of CIN. In addition, we assessed serial changes in serum creatinine within 5 days, serum cystatin-C within 48 hours and 8-isoprostane serum levels within 48 hours. Secondary indices of outcome were the need for RRT for a 10-day period following the infusion of the contrast agent, ICU stay and mortality.

Assuming that the incidence of CIN is 50% [25] we estimated that a sample size of 58 patients per treatment group would be required to detect 50% relative reduction in the incidence of CIN in the NacA with 80% power and 95% confidence level.

### Statistical analysis

All analysis was performed on an intention-to-treat basis. The results are expressed as means ± standard error (SE) unless otherwise stated. Data were compared between groups using Fisher’s exact test for categorical variables and the *t* test or Mann–Whitney test as appropriate for continuous variables. Differences in changes in serum creatinine, cystatin-C and 8-isoprostane concentrations (dependent variables) during time were analyzed by linear mixed model analysis. The kinetic of serum creatinine, cystatin-C and 8-isoprostane are indicated by mean regression lines. The slope of the regression line is the rate at which the examined parameter’s value changes day after day. The intercept of the regression line represents the value of the *y* (dependant variable) axis where the mean regression line crosses the *y* axis at theoretical day 0. Receiver operating characteristic (ROC) analysis was performed to illustrate the performance of clinical or laboratory variables in identifying patients with CIN. Only variables that were associated with CIN in univarate analysis were used in ROC analysis. *P* values <0.05 were considered to be statistically significant. Statistical analysis and preparation of graphs were performed using the statistical package SPSS 21.0 (SPSS Inc., Chicago, IL, USA), and GraphPad Prism (version 5.01).

## Results

**Fig. 1:**
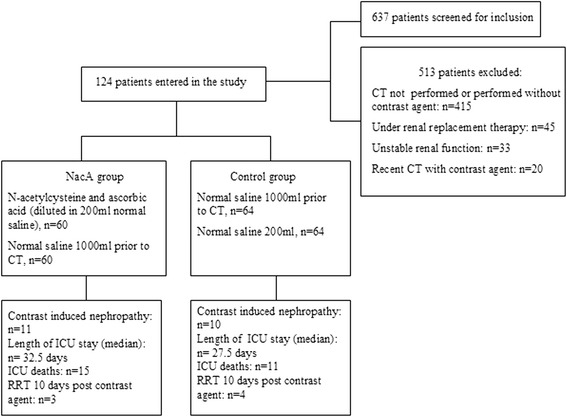
Flowchart for the study population. NacA, treatment group; ICU, intensive care unit; RRT, renal replacement therapy; CT, computed tomography

There were 124 patients who participated in the study, with 64 patients in the CG and 60 patients in the NacA (Fig. 1). Table 1 and Additional file 1: Tables S1 and S2 represent characteristics of participants at the entry of the study. There was a trend towards a larger percentage of patients with diabetes mellitus to have been included in the NacA, and for patients in the NacA to have received more contrast agent, whereas patients in the CG tended to have received more fluids (Table 2). Length of ICU stay and mortality were not significantly different between the two groups. Additional file 1: Table S3 indicates significant characteristics in univariate analysis of survival. Mortality was independently associated only with age (odds ratio, 1.06; 95% CI, 1.0–1.13) in multivariate analysis.

**Table 1:** Characteristics of participants at study entry

	All patients (*n* = 124)	NacA group (*n* = 60)	Control group (*n* = 64)	*P* value
Age (years)	50.90 (1.89)	51.34 (2.71)	50.51 (2.65)	0.82
Male gender, *n* (%)	96 (77.41)	46 (76.66)	55 (85.93)	0.24
Weight (Kg)	70.41 (2.42)	70.17 (3.07)	70.7 (4.01)	0.91
Height (cm)	168 (0.88)	165 (1.18)	169 (1.27)	0.40
BMI (Kg/m^2^)	27.41 (1.36)	25.63 (1.85)	29.63 (1.83)	0.59
APACHE II score	13.85 (0.63)	14.44 (1.01)	13.29 (0.78)	0.37
SOFA score	6.04 (0.32)	5.94 (0.5)	6.14 (0.41)	0.75
Category of admission				
Medical, *n* (%)	53 (42.74)	23 (38.33)	30 (46.87)	0.36
Surgical, *n* (%)	29 (23.38)	13 (21.66)	16 (25)	0.67
Neurosurgical, *n* (%)	42 (33.87)	24 40)	18 (28.12)	0.18
Comorbidities				
Diabetes mellitus, *n* (%)	12 (9.67)	9 (15)	3 (4.68)	0.06
Hypertension, *n* (%)	36 (29.03)	18 (30)	18 (28.12)	0.84
Cardiovascular, *n* (%)	14 (11.29)	8 (6.45)	6 (9.37)	0.57
Cirrhosis, *n* (%)	1 (0.80)	1 (1.6)	0	0.48
History of renal disease, *n* (%)	10 (8.06)	6 (10)	4 (6.25)	0.52
Sepsis, *n* (%)	43 (34.67)	24 (40)	19 (29.68)	0.57
COPD, *n* (%)	19 (15.32)	10 (16.66)	9 (14.06)	0.80
Charlson index score	2.15 (0.22)	2.47 (0.38)	1.86 (0.24)	0.17
Serum creatinine (mg/dl)	0.79 (0.06)	0.81 (0.10)	0.75 (0.06)	0.10
Serum cystatin-C (mg/L)	0.92 (0.06)	0.97 (0.07)	0.92 (0.09)	0.78
Serum urea,median (mg/dl)	45 (3.48)	56 (5.51)	41 (4.22)	0.08
Urea/creatinine	61.39 (2.41)	60.21 (3.51)	62.69 (3.25)	0.60
Fluid balance/24 h (ml)	1388 (151.7)	1133 (194.8)	1633 (227.6)	0.09
Vasoactive therapy, *n* (%)	45 (36.29)	20 (33.33)	25 (39.06)	0.57
Noradrenaline dose, γ	0.65 (0.02)	0.13 (0.03)	0.09 (0.03)	0.46
Mechanical ventilation, *n* (%)	76 (61.29)	32 (53.33)	44 (68.75)	0.09
Diuretic therapy, *n* (%)	19 (15.32)	12 (20)	7 (10.93)	0.21
ACEi or ARBs, *n* (%)	27 (21.77)	14 (23.33)	13 (20.31)	0.82
Nephrotoxic medications, *n* (%)	87 (70.16)	43 (71.66)	44 (68.75)	0.82
CIN, *n* (%)	21 (16.93)	11 (18.33)	10 (15.62)	0.81
Volume of contrast agent (ml)	123 (2.50)	128.75 (3.95)	119 (3.16)	0.06
Multiple studies, *n* (%)	19 (15.32)	6 (10)	13 (20.31)	0.62
Length of ICU stay before entering the study (days)	18.61 (2.4)	15.74 (2.3)	21.68 (4.07)	0.18
Length of ICU stay (days, median)	28.5 (3.53)	32.5 (5.11)	27.5 (4.49)	0.34
ICU mortality, *n* (%)	26 (20.96)	15 (25)	11 (17.18)	0.37
RRT 10 days post contrast agent, number of patients (%)	7 (5.64)	3 (5)	4 (6.25)	1.00

Data are presented as mean (SE) unless otherwise indicated. Nephrotoxic drugs included aminoglycosides, amphotericin, colimycin, vancomycin, teicoplanin and any non-steroidal anti-inflammatory drug (at least one)

*NacA group* Treatment (N-acetylcysteine and ascorbic acid) group, *BMI* body mass index, *APACHE* Acute Physiology and Chronic Health Evaluation, *SOFA* Sequential Organ Failure Assessment, *COPD* chronic obstructive pulmonary disease, *ACEi* angiotensin-converting enzyme inhibitor, *ARBs* angiotensin II receptor blockers, *RRT* renal replacement therapy

**Table 2:** Fluid balance of patients included in the study according to treatment group

Fluid balance	All patients (*n* = 124)	NacA group (*n* = 60)	Control group (*n* = 64)	*P* value
Before (2 days) contrast infusion	1145.6 (178.2)	1000.9 (266.3)	1269.0 (244.2)	0.46
Before (1 day) contrast infusion	1399.7 (151.0)	1117.5 (198.4)	1655.9 (225.3)	0.08
Day of contrast infusion	1463.6 (159.4)	1133 (194.8)	1633.4 (227.6)	0.09
First day after contrast infusion	1310.4 (167.1)	1317.1 (256.4)	1306.3 (224.3)	0.97
Second day after contrast infusion	996.1 (137.6)	1095.6 (237.8)	945.3 (163.0)	0.59
Third day after contrast infusion	844.9 (174.2)	902.9 (298.2)	774.4 (195.8)	0.71
Fourth day after contrast infusion	869.8 (167.4)	1042.5 (241.2)	691.9 (236.8)	0.30
Fifth day after contrast infusion	897.6 (235.5)	870.3 (340.3)	967.4 (234.3)	0.70

Data presented as mean (SE)

*NacA group* Treatment (N-acetylcysteine and ascorbic acid) group

### CIN

The incidence of CIN in the CG and NacA was 15.6% and 18.33%, respectively (*p* = 0.81). There was no significant difference between the two groups in serum creatinine during the examination period. Table 3 shows characteristics of participants according to the presence of CIN or not. Additional file 1: Table S4 shows a classification of patients with CIN based on serum creatinine or cystatin-C changes or the Risk, Injury, Failure, Loss, End-Stage Renal Failure (RIFLE) score. Compared to patients without CIN, patients with CIN had significantly increased baseline values of urea/creatinine ratio (*p* = 0.01) and had more often received colimycin or at least one of the following drugs that have a known adverse impact in renal function: aminoglycosides, amphotericin, colimycin, vancomycin, teicoplanin or non-steroid anti-inflammatory drugs (Additional file 1: Table S5). Additional file 1: Table S6 shows fluid balance in patients with or without CIN. Multivariate analysis revealed that CIN was associated with a higher baseline ratio of serum urea/creatinine (odds ratio, 1.02; 95 CI%, 1.00–1.05) and with the concomitant use of nephrotoxic medications (odds ratio, 0.24; 95 CI%, 0.06–0.94), or with a higher baseline ratio of serum urea/creatinine (odds ratio, 1.03; 95 CI%, 1.00–1.05) and with the concomitant use of colimycin (odds ratio, 0.25; 95 CI%, 0.08–0.78) as independent risk factors. CIN did not have significant impact on ICU mortality (28.57% versus 19.42%) or on ICU stay (29 versus 25 days) (Table 3).

**Table 3:** Characteristics of participants at study entry, with or without contrast-induced nephropathy (CIN)

	CIN (*n* = 21)	No CIN (*n* = 103)	*P* value
Age (years)	54 (4.90)	50.80 (2.12)	0.55
Male gender, *n* (%)	17 (80.95)	79 (76.69)	0.78
Weight (Kg)	73.23 (2.32)	68.15 (1.34)	0.68
Height (cm)	164	169	0.57
BMI (Kg/m^2^)	27	26	0.80
APACHE II score	14.95 (1.79)	13.68 (0.69)	0.51
SOFA score	6.53 (0.8)	5.69 (0.34)	0.34
Category of admission			
Medical, *n* (%)	9 (42.86)	44 (42.72)	0.15
Surgical, *n* (%)	2 (9.52)	27 (26.21)	0.15
Neurosurgical, *n* (%)	10 (47.62)	32 (31.07)	0.20
Comorbidities			
Diabetes, *n* (%)	2 (9.52)	10 (9.71)	0.68
Hypertension, *n* (%)	4 (19.05)	32 (31.07)	0.57
Cardiovascular, *n* (%)	3 (14.29)	11 (10.68)	0.70
Cirrhosis, *n* (%)	1 (4.76)	0	0.16
Renal disease, *n* (%)	1 (4.76)	6 (5.83)	0.41
Sepsis, *n* (%)	8 (38.10)	35 (33.98)	0.80
COPD, *n* (%)	3 (14.28)	16(15.53)	1.00
Charlson index score	2.32 (0.57)	2.10 (0.24)	0.70
Serum creatinine (mg/dl)	0.87 (0.08)	0.96 (0,07)	0.39
Serum cystatin-C (mg/L)	1.34 (0.23)	0.98 (0.05)	0.14
Serum urea (mg/dl, median)	55 (8.34)	44 (3.82)	0.18
Serum urea/creatinine	73.19 (6.3)	58.65 (2.5)	0.01
Fluid balance/24 h (ml)	1462 (169)	1058 (338.1)	0.30
Vasoactive therapy, *n* (%)	9 (42.86)	45 (43.69)	0.13
Noradrenaline dose, γ	0.1 (0.05)	0.06 (0.01)	0.20
Mechanical ventilation, *n* (%)	15 (71.43)	51 (49.51)	0.09
Diuretic therapy, *n* (%)	1 (4.76)	19 (18.45)	0.19
ACEi or ARBs, *n* (%)	7 (33.33)	20 (19.42)	0.19
Nephrotoxic medications, *n* (%)	19 (90.48)	68 (66.02)	0.03
Volume of contrast agent (ml)	126.3 (5.88)	122.2 (2.77)	0.53
Multiple studies, *n* (%)	6 (28.57)	13 (12.62)	0.09
Length of ICU stay (days, median)	29 (3.97)	25 (7.85)	0.80
Length of ICU stay before entering the study (days)	20.71 (4.4)	18.15 (2.7)	0.68
ICU mortality, *n* (%)	6 (28.57)	20 (19.42)	0.38
RRT 10 days post contrast agent, *n* (%)	3 (14.28)	4 (4.85)	0.09
Total duration on RRT (h)	47.67 (17.32)	49.25 (9.29)	0.93

Data presented as mean (SE) unless otherwise indicated. Nephrotoxic drugs included at least one of the following: aminoglycosides, amphotericin, colimycin, vancomycin, teicoplanin or any non-steroidal anti-inflammatory drug

*CIN* contrast-induced nephropathy, *BMI* body mass index, *APACHE* Acute Physiology and Chronic Health Evaluation, *SOFA* Sequential Organ Failure Assessment score, *COPD* chronic obstructive pulmonary disease, *ACEi* angiotensin converting enzyme inhibitor, *ARBs* angiotensin II receptor blockers, *RRT* renal replacement therapy

### Serum cystatin-C

**Fig. 2:**
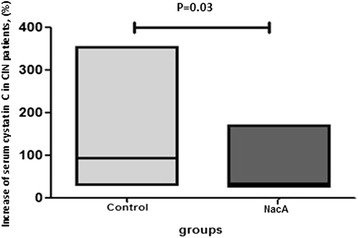
Serum cystatine C levels changes (%) between baseline and time of radio contrast induced nephropathy (CIN) diagnosis in controls and in patients who received antioxidants (NacA group). The horizontal lines in the low-high bar graphs represent median values. The statistical significance is indicated with the capped line

Serum cystatin-C (sCysC) concentration did not present significant differences between groups or during the examination period (*p* = 0.658). Patients in the CG who had CIN had significantly increased cystatin-C levels compared to patients with NacA who had CIN (*p* = 0.03) (Fig. 2). The percentage change (median ± interquartile range (IR)) in sCysC (mg/L) from baseline in patients who had CIN was 37.23% ± 12.54 and 93.20% ± 33.54 in NacA and in CG, respectively (*p* = 0.03). Data were also analyzed according to 25% increase of cystatin-C levels from the baseline value (25% DeltaCystC) - similarly to creatinine-based definition of CIN, but there was no significant difference between the NacA and CG (*p* = 0.26).

### Serum 8-isoprostane

**Fig. 3:**
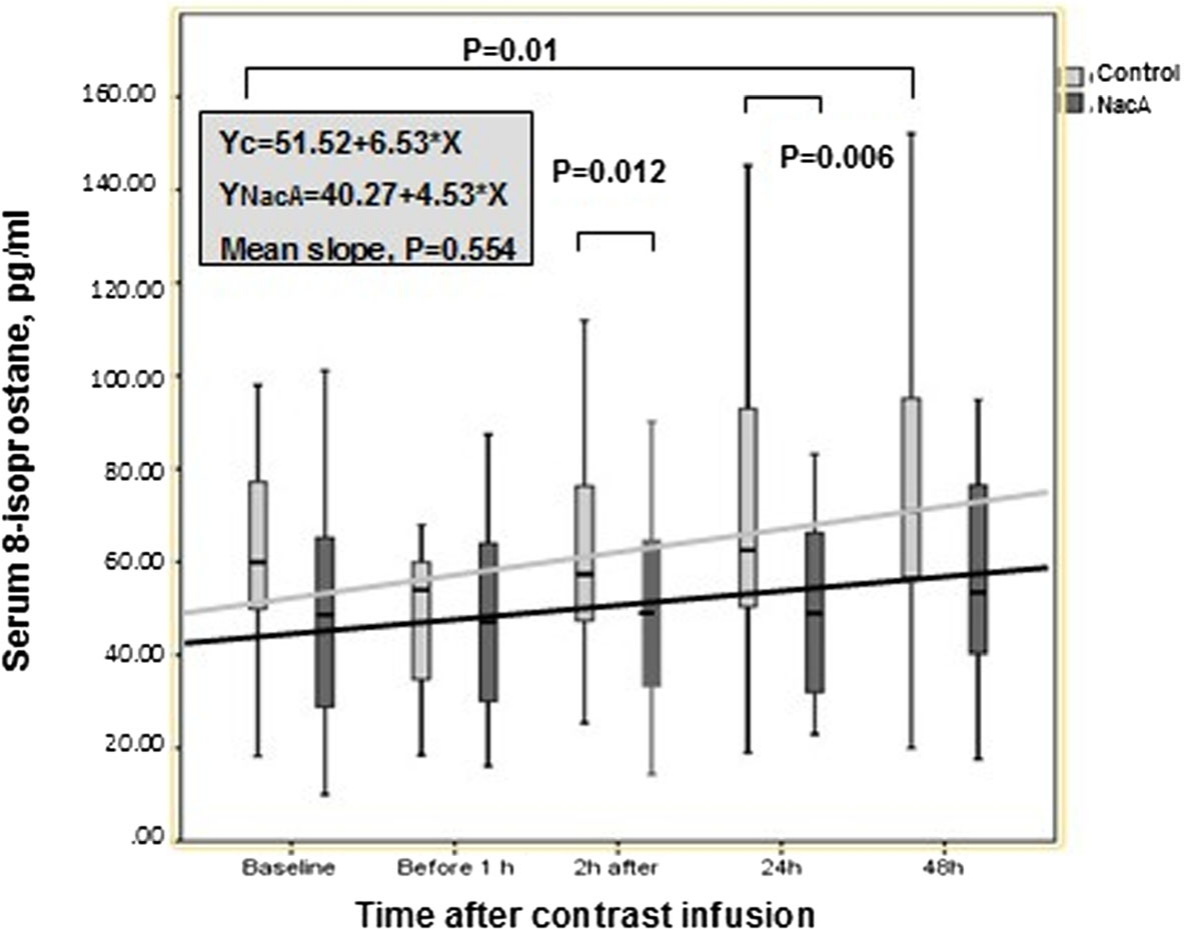
Serum 8-isoprostane levels changes during 48 hours following administration of radio contrast material in controls and in patients who received antioxidants (NacA group). The significant differences are marked with brackets. Forty-eight hours kinetics is indicated by the corresponding mean regression lines

Serum levels of 8-isoprostane are presented in Fig. 3. Although the mean regression lines of serum levels of 8-isoprostane did not differ significantly between the two groups over time, significant differences between the NacA and CG were detected at 2 and 24 hours after the infusion of the contrast agent and in the CG between baseline and 48 hours.

### Diagnostics

**Fig. 4:**
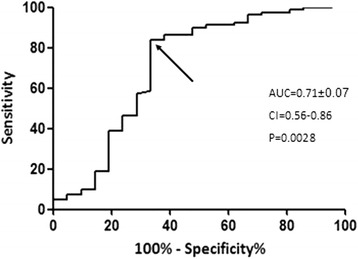
Diagnostic performance of baseline values of the serum urea/creatinine ratio in predicting contrast-induced nephropathy

**Fig. 5:**
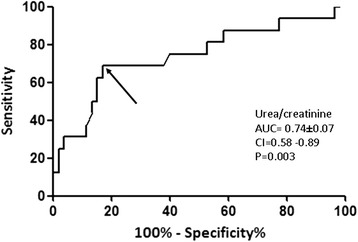
Diagnostic performance of baseline values of the serum urea/creatinine ratio in predicting contrast-induced nephropathy in patients who received nephrotoxic medications

ROC curve analysis showed that the best cutoff baseline value of the urea/creatinine ratio to predict CIN was 63.79 (area under the curve (AUC) (95% CI) 0.712 ± 0.076 (0.56–0.86), *p* = 0.002) (Fig. 4). ROC curve analysis also showed that the best cutoff baseline value of the serum urea/creatinine ratio to predict CIN in a patient who receives nephrotoxic medication was 65.70 (sensitivity 68.75%, specificity 69.81%, AUC (95%CI) 0.74 ± 0.078 (0.58–0.89), *p* = 0.003) (Fig. 5).

## Discussion

The main findings of the present study are: (a) intravenous infusion of the combination of two antioxidant agents, N-acetylcysteine and ascorbic acid, failed to reduce the incidence of CIN; (b) CIN was associated with increased baseline values of the serum urea/creatinine serum ratio and the concomitant use of nephrotoxic medications; (c) antioxidants may have reduced oxidative stress as indicated by 8-isoprostane serum levels, which were significantly lower in the NacA compared to the CG at 2 hours and 48 hours; (d) in patients with CIN in the NacA, serum cystatin-C values increased less than in patients with CIN in the CG.

The pathogenesis of CIN is considered multifactorial, with renal vasoconstriction and direct cell toxicity, which both lead to medullary hypoxia and the production of reactive oxygen species [21, 26]. The co-administration of the antioxidant agents could help in managing the potential oxidative burden added by the radiocontrast material. Yet, there was no significant difference in the incidence of CIN between the NacA and controls. Several explanations could be given for the absence of a difference between groups. Critically ill patients present with many predisposing factors for deterioration of renal function. In this respect it might be difficult to assess the net impact of the contrast agent in renal function [27]. Individual cases of nephropathy occurring in critically ill patients after use of intravenous radiocontrast material cannot be attributed with certainty to the contrast exposure. In this respect, the definition of CIN is challenging and the incidence of CIN presents varies greatly between studies, i.e. from 1.5 to 33% [13, 14]. In the present study, we included renally stable patients and the definition of CIN was based on change in serum creatinine, an index that is widely accepted and available [28]. The incidence of CIN was 15.6%.

Another point that could be noted to help explain the absence of a difference between NacA and CG was that there were indications that CG included fewer patients with diabetes mellitus or that patients in the NacA received higher volumes of radiocontrast material (Table 1) or that patients in the CG received more fluids at 1 day before and on the day of radiocontrast material infusion (Table 2). These factors might have obscured any significant prophylactic impact of the antioxidant agents on renal function.

In order to provide further insight into the relationship between CIN and potential risk factors, we analyzed the impact of several clinical factors associated with renal deterioration. We found that CIN was associated with the concomitant use of nephrotoxic medications (*p* = 0.03). This has been underlined by previous studies on the etiology of CIN in critically ill patients [29]. Notably in this study, we found that colimycin was related to CIN, which underlines the adverse impact of this antibiotic, which is unfortunately often necessary for management of gram-negative infections nowadays.

Other well-known etiologic factors for CIN such as co-morbidities (i.e. diabetes mellitus or preexisting renal failure) were not associated with CIN. This is in accordance with other studies [29, 30] addressing CIN risk factors in ICU patients. A possible explanation for this fact may be related to the great heterogeneity of critically ill patients or to the population size in this study, which could not depict such differences. We should also underline here that the present study did not include a special strategy for fluids administration/balance and all relevant decisions were based on treating physicians’ decisions, which were according to current clinical practice recommendations [22]. Yet, we found no difference in fluid balance between groups.

In this study the main outcome was CIN based on serum creatinine values. One might argue that creatinine may not be a sensitive index of glomerular filtration rate (GFR) alterations [31]. Previous studies suggested that creatinine metabolism can be affected by Nac, so that the observed changes in serum creatinine concentration after administration of Nac may not be indicative of GFR improvement [32]. In order to overcome these obstacles we assessed sCysC concentration, which can serve as an endogenous marker of renal function and is believed to be superior to plasma creatinine concentration as it does not depend on age, sex and muscle mass, and so it has been considered as a simple, reliable and accurate marker of renal function [33, 34]. Thus, we identified patients with 25% DeltaCysC - similarly to creatinine-based definition of CIN. Although no significant difference was depicted, more patients presented with severe CIN (increase of more than 50% from baseline values) in the CG compared to the NacA. We acknowledge that other sensitive markers such as urine neutrophil gelatinase-associated lipocalin (NGAL) [35] could be more accurate to define the impact of CIN; however, this was not studied in this investigation and might be the aim of a future study in ICU patients.

Despite the fact that this strategy failed to reduce the incidence of CIN, or other indices of outcome we found evidence that it may at least partially balance the oxidative stress burden, as patients in the NacA had lower 8-isoprostane levels following radiocontrast material infusion and patients with CIN had attenuation of the increase in serum cystatin-C levels. Serum levels of 8-isoprostane represent a sensitive index of oxidative stress in vivo [36]. In this study, although the overall kinetics of serum levels of 8-isoprostane did not differ between the two groups, as indicated by the mean regression lines, significant differences between the two groups were detected at 2 hours and 24 hours after the infusion of antioxidant agents, and in the CG between baseline and 48 hours (Fig. 3).

## Conclusion

In the present study the use of the combination of antioxidant agents, Nac and Aa, failed to reduce the incidence of CIN in critically ill patients undergoing CT with radiocontrast material. Furthermore, our findings indicate that an increased urea/creatinine ratio and the use of nephrotoxic medications, especially of colimycin, may have an adverse impact on renal function in this setting. Despite the absence of a significant impact of Nac-Aa in the incidence of CIN, the use of antioxidants partially balanced the oxidative stress burden following contrast infusion and decreased renal injury, as it was assessed using serum cystatin-C in patients who presented with CIN. In this respect, our results should be validated in a large number of patients in a multi-center randomized clinical trial in the future.

## Additional file

Additional file 1: Table S1. Classification of participants renal function according to RIFLE criteria at the entry study. **Table S2.** Medications with potential impact on renal function received by participants according to treatment group. **Table S3.** Univariate analysis of characteristics of survivors and non survivors. **Table S4.** CIN based on serum creatinine or cystatin-C changes or RIFLE score (between the day of radiocontrast material infusion and the day of CIN diagnosis). **Table S5.** Medications with potential impact on renal function received by participants according to the presence of CIN or not. **Table S6.** Fluid balance of patients included in the study according to the presence of CIN or not. (DOC 110 kb)

## Abbreviations

Aa: Ascorbic acid
ACEi: Angiotensin converting enzyme inhibitor
APACHE II: Acute Physiology and Chronic Health Evaluation
ARBs: Angiotensin II receptor blockers
AUC: Area under the curve
BMI: Body mass index
CG: Control group
CI: Confidence interval
CIN: Contrast-induced nephropathy
COPD: Chronic obstructive pulmonary disease
CT: Computed tomography
ESRD: End-stage renal disease
GFR: Glomerular filtration rate
ICU: Intensive care unit
IR: Interquartile range
N/S: Normal saline
Nac: N-acetylcysteine
NacA: Treatment group
RIFLE: Risk Injury Failure Loss End-Stage Renal Failure
ROC: Receiver operating characteristic
RRT: Renal replacement therapy
sCysC: Serum cystatin-C
SE: Standard error
SOFA: Sequential Organ Failure Assessment
