# Supplementary material for: The impact of N-acetylcysteine and ascorbic acid in contrast-induced nephropathy in critical care patients: an open-label randomized controlled study
Source: Crit Care. 2017 Oct 31;21:269. doi: 10.1186/s13054-017-1862-3 (PMC5664844; doi:10.1186/s13054-017-1862-3)
Supplement: Additional file 1: Table S1. — Classification of participants renal function according to RIFLE criteria at the entry study. Table S2. Medications with potential impact on renal function received by participants according to treatment group. Table S3. Univariate analysis of characteristics of survivors and non survivors. Table S4. CIN based on serum creatinine or cystatin-C changes or RIFLE score (between the day of radiocontrast material infusion and the day of CIN diagnosis). Table S5. Medications with potential impact on renal function received by participants according to the presence of CIN or not. Table S6. Fluid balance of patients included in the study according to the presence of CIN or not. (DOC 110 kb) [file 13054_2017_1862_MOESM1_ESM.doc]

**Table S1:** Classification of participants renal function according RIFLE criteria at the entry study.

|  | **NacA Group**  **(n=60)** | **Control Group**  **(n=64)** | **P value** |
| --- | --- | --- | --- |
|  |  |  |  |
| Normal, n(%) | 56(93.33) | 62(96.87) | 0.48 |
| Risk | 2(3.33) | 2(3.12) | 1.0 |
| Injury | 1(1.6) | 0 | 0.48 |
| Failure | 1(1.6) | 0 | 0.48 |
| Loss | 0 | 0 | - |
| ESRD | 0 | 0 | - |

ESRD: End stage renal disease.

| **Table S2:** Medications with potential impact in renal function received by participants according to treatment group. | | | | |
| --- | --- | --- | --- | --- |
|  | **All patients**  **(n=124)** | **NacA Group**  **(n=60)** | **Control Group**  **(n=64)** | **P value** |
| Aminoglycosides, n(%) | 14(11.29) | 6(10) | 8(12.5) | 0.77 |
| Colimycin, n(%) | 44(35.48) | 13(21.66) | 19(29.68) | 0.41 |
| Vancomycin, n(%) | 10(8.06) | 7(11.67) | 3(4.69) | 0.19 |
| Teicoplanine, n(%) | 9(7.26) | 5(8.33) | 7(10.94) | 0.76 |
| Amphotericine, n(%) | 5(4.03) | 3(5) | 2(3.13) | 0.67 |
| Non steroidal anti-inflammatory, n(%) | 26(20.97) | 16(26.67) | 10(15.63) | 0.18 |
| ACEI or ARBs, n(%) | 27(21.77) | 14(23.33) | 13(20.31) | 0.82 |
| B-blockers, n(%) | 28(22.58) | 15(25) | 13(20.31) | 0.66 |
| Statines, n(%) | 14(11.29) | 7(11.67) | 7(10.94) | 1.00 |
| Diuretics, n(%) | 19(15.32) | 12(20) | 7(10.93) | 0.21 |
| Nephrotoxic medications used*, n(%) | 87(70.16) | 43(71.66) | 44(68.75) | 0.84 |
| Nephrotoxic medications/participant, n(%) | 1.03 | 1.02 | 1.04 | 0.91 |
| Data are presented as n(%) otherwise is indicated.  ACEi: angiotensin converting enzyme inhibitor; ARBs: angiotensin II receptor blockers  Nephrotoxic medications included: *(at least one of the following medications) aminoglycosides, colimycin, , vancomycin, teicoplanin, amphotericin and any non steroidal anti-inflammatory medication. | | | | |

| **Table S3:** Univariate analysis of characteristics of survivors and non survivors. | | | |
| --- | --- | --- | --- |
|  | **Survivors**  **(n=98)** | **Non survivors (n=26)** | **P value** |
| Male gender, n(%) | 79(82.29) | 19(19.79) | 0.42 |
| Age (years) | 47.64(2.22) | 62.59(2.92) | 0.001 |
| Weight (Kgrs) | 70.2(2.7) | 68.3(4.4) | 0.97 |
| BMI (Kgrs/m^2^) | 26.77(0.84) | 26.90(1.5) | 0.93 |
| CHARLSON index score | 1.77(0.37) | 3.63(0.58) | 0.14 |
| APACHE II score (at admission) | 13.07(0.56) | 16.83(1.22) | 0.002 |
| SOFA score (at admission) | 7(0.36) | 8.08(0.76) | 0.15 |
| APACHE II score (day of radio contrast material infusion) | 12.84(0.66) | 17.25(1.61) | 0.004 |
| Diabetes mellitus, n(%) | 9(9.18) | 0.0 | 1.0 |
| Serum urea (at baseline, mg/dl) | 42.25(5.9) | 59.75(75) | 0.02 |
| Serum creatinine (mg/dl, at baseline) | 1.07(0.12) | 0.83(0.09) | 0.85 |
| Serum cystatin-C (at baseline, mg/L) | 0.77(0.12) | 1.0(0.12) | 0.37 |
| Serum urea/creatinine | 56.23(2.48) | 75.5(5.08) | 0.0001 |
| Nephrotoxic medications, n(%) | 61(62.24) | 26(100) | 0.38 |
| Nephrotoxic medications/ participant, n(%) | 0.77(0.11) | 1(0.20) | 0.78 |
| Fluid balance, ml | 1337.9(187.3) | 1259.3(242.3) | 0.50 |
| Noradrenaline, γ | 0.05(0.01) | 0.22(0.1) | 0.01 |
| APACHE II score (day of radio contrast material infusion) | 12.84(0.66) | 17.25(1.61) | 0.004 |
| SOFA score (day of radio contrast material infusion) | 5.57(0.44) | 8.38(0.8) | 0.006 |
| Length of ICU stay before entering the study, days | 18.29(3) | 19.46(3.5) | 0.99 |
| NacA Group, n(%) | 45(45.92) | 15(57.69) | 0.37 |
| Radiocontrast material infused (ml) | 120.45(3.7) | 137.5(5.5) | 0.006 |
| Nephropathy post radiocontrast material infusion, n(%) | 15(15.31) | 6(23.08) | 0.38 |
| Length of ICU stay(, days) | 38.41(4.4) | 44.77(6.1) | 0.25 |
| Data presented as mean (standard error), BMI: Body mass index; APACHE: Acute Physiology and Chronic Health Evaluation; SOFA: Sequential Organ Failure Assessment score; Nephrotoxic medications included: aminoglycosides, amphotericin, colimycin, vancomycin, teicoplanin, and any non-steroidal anti-inflammatory medication (at least one). | | | |

| **Table S4:** CIN based on serum creatinine or cystatin-C changes or RIFLE score (between the day of radio contrast material infused and the day of CIN diagnosis). | | | |
| --- | --- | --- | --- |
|  | **NacA Group**  **(n=60)** | **Control Group**  **(n=64)** | **P value** |
|  |  |  |  |
| CIN based on serum creatinine changes, n(%) | 11(18.33) | 10(15.6) | 0.81 |
| - Increase by 25- 50%, n(%) | 9(81.81) | 5(50) |  |
| - Increase by 50-100%, n(%) | 2(18.18) | 1(10) |  |
| - Increase by 100-200%, n(%) | 0 | 2(20) |  |
| - Increase >200%, n(%) | 0 | 1(10) |  |
|  |  |  |  |
| CIN based on serum cystatin-C changes, n(%). | 12(20) | 10(15.62) | 0.63 |
| - Increase by 25- 50%, n(%) | 8(66.66) | 2(20) |  |
| - Increase by 50-100%, n(%) | 3(12) | 3(30) |  |
| - Increase by 100-200%, n(%) | 1(8.33) | 3(30) |  |
| - Increase >200%, n(%) | 0 | 2(20) |  |
|  |  |  |  |
| CIN based on RIFLE criteria, n(%) | 6(10) | 8(12.5) | 0.77 |
| - Risk, n(%) | 3(50) | 7(87.5) |  |
| - Injury, n(%) | 2(33.33) | 1(12.5) |  |
| - Failure, n(%) | 1(16.66) | 0 |  |
| - Loss, n(%) | 0 | 0 |  |
| - ESRD, n(%) | 0 | 0 |  |
|  |  |  |  |
| | Data represent number of patient n(%). | | --- |   CIN: Contrast induced nephropathy, ESRD: End stage renal disease. | | | |

**Table S5**: Medications with potential impact in renal function received by participants according to the presence of CIN or not.

|  | **CIN**  **(n=21)** | **No CIN**  **(n=103)** | **P value** |
| --- | --- | --- | --- |
| Aminoglycosides, n(%) | 4(19.04) | 10(9.70) | 0.25 |
| Colimycin, n(%) | 12(57.14) | 32(31.06) | 0.04 |
| Vancomycin, n(%) | 3(14.28) | 7(6.79) | 0.37 |
| Teicoplanin, n(%) | 2(9.52) | 7(6.79) | 0.64 |
| Amphotericin, n(%) | 2(9.52) | 3(2.91) | 0.19 |
| Non steroidal anti-inflammatory, n(%) | 6(28.57) | 20(19.42) | 0.38 |
| ACEI or ARBs, n(%) | 7(33.33) | 20(19.42) | 0.24 |
| B-blockers, n(%) | 3(14.28) | 25(24.27) | 0.40 |
| Statins, n(%) | 2(9.52) | 12(11.65) | 1.00 |
| Diuretics, n(%) | 1(4.76) | 19(18.45) | 0.19 |
| Nephrotoxic medications used*, n(%) | 19(90.48) | 68(66.02) | 0.03 |
| Nephrotoxic medications/participant, n | 1.33(0.16) | 0.96 | 0.06 |
| Data are presented as n(%) otherwise is indicated.  ACEi: angiotensin converting enzyme inhibitor; ARBs: angiotensin II receptor blockers.  Nephrotoxic medications included: *(at least one of the following  medications) aminoglycosides, colimycin, , vancomycin, teicoplanin,  amphotericin and any non steroidal anti-inflammatory. | | | |

**Table S6:** Fluid balance of patients included in the study according to the presence of CIN or not.

|  | **CIN**  **(n=21)** | **No CIN**  **(n=103)** | **P value** |
| --- | --- | --- | --- |
|  |  |  |  |
| 2 days before contrast infusion | 1183.4(570.6) | 1168.5(191.9) | 0.97 |
| 1 day before contrast infusion | 1364.4(472.7) | 1412.4(164.3) | 0.90 |
| Day of contrast infusion | 1462(169) | 1058(338.1) | 0.30 |
| 1st day after contrast infusion | 1214.1(420.9) | 1667.5(181.8) | 0.29 |
| 2^nd^ day after contrast infusion | 1077.9(306.4) | 1437.2(201.3) | 0.43 |
| 3^rd^ day after contrast infusion | 1237.0(433.8) | 984.8(143.2) | 0.50 |
| 4^th^ day after contrast infusion | 1479.4(384.4) | 770.3(204.2) | 0.14 |
| 5^th^ day after contrast infusion | 1373.7(349.8) | 749.5(201.2) | 0.19 |
| Data presented as mean (standard error). CIN: contrast induced  nephropathy. | | | |
